# Comparison of Injury Severity Score (ISS) and New Injury Severity Score (NISS) in the Evaluation of Thoracic Trauma Patients: A Retrospective Cohort Study

**DOI:** 10.1155/2024/4861308

**Published:** 2024-08-23

**Authors:** He Jin, Yuanyuan Zhang, Qi Zhang, Lijuan Ouyang, Xueyao Li, Yiyan Zhang, Baosheng Yang, Junfeng Sun, Chaohui Wei, Guimei Yang, Li Guan, Shilan Luo, Junyu Zhu, Huaping Liang

**Affiliations:** ^1^ Department of Cardiothoracic Surgery 926th Hospital of Joint Logistics Support Force of PLA, Kaiyuan 661600, Yunnan, China; ^2^ Department of Wound Infection and Drug Daping Hospital Army Medical University State Key Laboratory of Trauma and Chemical Poisoning, Chongqing 400042, China; ^3^ Department of Disease Control and Prevention 926th Hospital of Joint Logistics Support Force of PLA, Kaiyuan 661600, Yunnan, China; ^4^ Department of Orthopedic Surgery 926th Hospital of Joint Logistics Support Force of PLA, Kaiyuan 661600, Yunnan, China; ^5^ Department of Burns and Plastic Surgery 926th Hospital of Joint Logistics Support Force of PLA, Kaiyuan 661600, Yunnan, China

## Abstract

**Objective:**

To explore the value of the injury severity score (ISS) and the new injury severity score (NISS) for evaluating injuries and predicting complications (pneumonia and respiratory failure) and poor prognoses (in-hospital tracheal intubation, extended length of hospital stay, ICU admission, prolonged ICU stay, and death) in patients with thoracic trauma.

**Methods:**

The data of consecutive patients with thoracic trauma who were admitted to the department of cardiothoracic surgery of a tertiary hospital between January 2018 and December 2021 were retrospectively collected. ISS and NISS were calculated for each patient. The study outcomes were complications and poor prognoses. The differences in ISS and NISS between patients with complications and poor prognoses and patients without the abovementioned conditions were compared using the Mann‒Whitney *U* test. Discrimination and calibration of ISS and NISS in predicting outcomes were compared using the area under the receiver operating characteristic (ROC) curve (AUC) and Hosmer‒Lemeshow (H-L) statistic.

**Results:**

A total of 310 patients were included. ISS and NISS of patients with complications and poor prognoses were greater than those of patients without complications and poor prognoses, respectively. The discrimination of ISS in predicting pneumonia, respiratory failure, in-hospital tracheal intubation, extended length of hospital stay, ICU admission, prolonged ICU stay, and death (AUCs: 0.609, 0.721, 0.848, 0.784, 0.763, 0.716, and 0.804, respectively) was not statistically significantly different from that of NISS in predicting the corresponding outcomes (AUCs: 0.628, 0.712, 0.795, 0.767, 0.750, 0.750, and 0.818, respectively). ISS showed better calibration than NISS for predicting pneumonia, respiratory failure, in-hospital tracheal intubation, extended length of hospital stay, and ICU admission but worse calibration for predicting prolonged ICU stay and death.

**Conclusion:**

ISS and NISS are both suitable for injury evaluation. There was no statistically significant difference in discrimination between ISS and NISS, but they had different calibrations when predicting different outcomes.

## 1. Introduction

Trauma is the leading cause of death and a major public health problem worldwide that results in 5.8 million deaths each year and substantial healthcare costs [1–4]. Thoracic trauma accounts for 10%–15% of all trauma cases and is responsible for approximately 25% of trauma-related deaths [5–8]. Injuries due to thoracic trauma affect the chest wall and internal organs of the thorax to different extents and may lead to complications such as pneumonia and respiratory failure, and patients with thoracic trauma frequently require admission to the intensive care unit (ICU) and mechanical ventilator support [9, 10]. Accurate evaluation of thoracic trauma severity in the early posttrauma phase is highly important for predicting complications and the prognosis, assessing the need for intensive care, and making the optimal clinical decision [11, 12]; therefore, accurate evaluation of thoracic trauma severity is the basis for effective treatment.

Trauma scoring systems have been developed to quantitatively evaluate trauma severity and have been used for predicting mortality and morbidity, triaging patients, stratifying the risks for research purposes, and benchmarking trauma outcomes [13–16]. Among the various trauma scoring systems, the injury severity score (ISS) is the most commonly used [17–19]. The ISS was proposed by Baker et al. [20] in 1974 based on the abbreviated injury scale (AIS). To calculate the ISS, the whole body is divided into six regions and the sum of the squares of the highest AIS values in each of the three most severely injured body regions is taken [20, 21]. Although the ISS has been widely used, an important limitation of the ISS is that the ISS does not account for more than one injury within the same body region [18, 19, 22]. To overcome this limitation, Osler et al. [23] introduced a simple modification of the ISS called the new injury severity score (NISS). The NISS is defined as the sum of the squares of the AIS values of the three most severe injuries regardless of the body region injured [23]. Numerous studies have compared the ability of the ISS and NISS to predict trauma outcomes in patients in a specific age group such as pediatric patients, patients with injuries in a specific body region such as head injuries, patients with injuries caused by a specific type of trauma such as penetrating trauma, and patients with injuries caused by a specific mechanism such as a firearm [13, 18, 24, 25]. To date, however, studies comparing the capacities of these two scoring systems in evaluating patients with thoracic trauma are rare, and the capabilities of the two scoring systems to predict different prognostic parameters in patients with thoracic trauma have not been fully elucidated.

The aim of this study was to validate the value of the ISS and NISS for injury evaluation and to compare the performance of these two scoring systems for the prediction of complications and poor prognoses, including pneumonia, respiratory failure, in-hospital tracheal intubation, extended length of hospital stay, ICU admission, prolonged ICU stay and death, in patients with thoracic trauma.

## 2. Methods

### 2.1. Ethics Approval

The study was approved by the Ethics Committee of 926th Hospital of Joint Logistics Support Force of PLA (no. 2022-126). Due to the anonymization of the data, the need for informed consent was waived.

### 2.2. Study Design

This study was conducted in a tertiary hospital in Yunnan Province, China. Consecutive patients with thoracic trauma who were admitted to the department of cardiothoracic surgery between 1 January 2018 and 31 December 2021 were retrospectively recruited in this research cohort. The inclusion criteria for this study were admission to the hospital within 24 hours of trauma and a hospital stay of 2 or more days. Patients younger than 16 years were excluded from the analyses.

### 2.3. Data Collection

Demographic data such as sex and age and injury characteristics including injury mechanism and the AIS value for each of the body regions injured were extracted from the registry. AIS values were coded using the AIS-2005. The ISS and NISS for each patient were then calculated as primary exposures in this study. Complications (pneumonia and respiratory failure) and poor prognoses (in-hospital tracheal intubation, extended length of hospital stay, ICU admission, prolonged ICU stay, and death) were examined from the registry as the study outcomes.

In this study, pneumonia is referred to as hospital-acquired pneumonia (HAP) and ventilator-associated pneumonia (VAP) [26]. The definitions for HAP and VAP from Chinese guidelines for the diagnosis and treatment of hospital-acquired pneumonia and ventilator-associated pneumonia in adults (2018 Edition) [27] were used as follows: HAP was defined as pneumonia not incubating at the time of hospital admission and occurring 48 hours or more after admission in patients not receiving invasive mechanical ventilation during hospitalization. VAP was defined as pneumonia occurring >48 hours after endotracheal intubation or tracheotomy to receive mechanical ventilation. Pneumonia occurring within 48 hours after weaning from mechanical ventilation and extubation was also considered VAP. A clinical diagnosis of pneumonia was established when the patient met the following criteria: a new or progressive infiltrate, consolidation, or ground glass opacity was revealed on chest radiograph or CT scan, plus two or more of the following three criteria: fever >38°C, purulent airway secretions, and peripheral white blood cell count of >10 × 10^9^/L or <4 × 10^9^/L [27]. Respiratory failure was defined as a decrease in arterial oxygen tension (PaO_2_) to less than 60 mmHg with or without an increase in arterial carbon dioxide tension (PaCO_2_) on room air [28]. In-hospital tracheal intubation was defined as a procedure involving the placement of a tube into the trachea during a hospital stay. Extended length of hospital stay was defined as a length of hospital stay of ≥10 days [19, 22]. ICU admission was defined as admission to the ICU during hospitalization. Prolonged ICU stay was defined as an ICU stay of >14 days [29]. Death was defined as all-cause death during a hospital stay.

### 2.4. Statistical Analyses

The primary objective of this study was to estimate the discrimination index, namely, the area under the receiver operating characteristic (ROC) curve (AUC). Assuming that the incidence of the study outcome was 20%, a sample size of 310 would produce a 95% confidence interval with a width of 0.149 (upper to lower bounds) and a targeted AUC of 0.75. If the incidence of the study outcome was 10%, the width would extend to 0.205.

The frequency and percentage of the qualitative variables were computed. The Kolmogorov–Smirnov test or the Shapiro–Wilk test was used to assess the normality of the continuous data. The mean and standard deviation of the data were calculated when the continuous data followed a normal distribution; otherwise, the median and interquartile range of the data were calculated. The Mann–Whitney *U* test was employed for statistical comparison of the data that were not normally distributed.

Measures of discrimination and calibration were used to compare the performance of the ISS and NISS in predicting pneumonia, respiratory failure, in-hospital tracheal intubation, extended length of hospital stay, ICU admission, prolonged ICU stay, and death. Discrimination indicates how well the model differentiates those at higher risk of having an event from those at lower risk, and calibration describes the accuracy of absolute risk estimates [30]. Discrimination was measured by the AUC and calibration was measured by the Hosmer‒Lemeshow (H-L) statistic [15]. An AUC of 1 indicates perfect discrimination, whereas an AUC of 0.5 indicates that the predictive ability of the model is no better than chance. The following scale is usually used to illustrate the predictive power represented by the AUC: 0.5 < AUC < 0.6 indicates failed prediction, 0.6 < AUC < 0.7 poor prediction, 0.7 < AUC < 0.8 fair prediction, 0.8 < AUC < 0.9 good prediction, and 0.9 < AUC < 1 excellent prediction [31]. A lower H-L statistic value indicates better calibration. The confidence intervals of the AUC were binomial exact confidence intervals; the confidence intervals of the cutoff and Youden index were estimated using the bias-corrected and accelerated (BCa) bootstrap method; the confidence intervals of the sensitivity and specificity were “exact” Clopper–Pearson confidence intervals; the confidence intervals of the likelihood ratios were calculated using the log method; and the confidence intervals of the predictive values were the standard logit confidence intervals.

A *p* value <0.05 was considered to be statistically significant. Statistical analysis was performed using SPSS version 20 and MedCalc version 20.

## 3. Results

**Figure 1:**
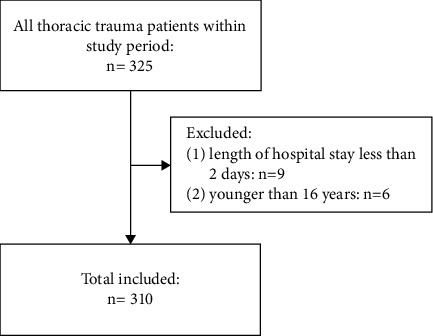
Overview of the patient inclusion process for the analysis.

A total of 310 thoracic trauma patients who met the inclusion criteria were enrolled in this study over a 4-year period (Figure 1). The descriptions of the patients included are shown in Table 1. There were 231 males (74.5%) and 79 females (25.5%). The mean age was 49.47 years (standard deviation = 12.15), ranging from 20 to 83 years. The median and interquartile range of the ISS and NISS were 13 (9, 17) and 17.5 (11, 22), respectively. The most frequent mechanism of injury was falls (56.1%), followed by traffic accidents (27.1%). The median and interquartile range of the length of hospital stay was 13 (8, 19) days. In the whole sample, 198 patients (63.9%) were admitted to the ICU. The median and interquartile range of the length of ICU stay of these 198 patients who were admitted to the ICU was 4 (2, 7) days. Overall, there were 4 deaths for a mortality rate of 1.3%.

The ISS and NISS of patients with complications (pneumonia and respiratory failure) and poor prognoses (in-hospital tracheal intubation, extended length of hospital stay, ICU admission, prolonged ICU stay, and death) were statistically greater than those of patients without complications and poor prognoses (Table 2).

The results of the ROC analysis of the two scoring systems, the ISS and NISS, for predicting the outcomes are shown in Table 3.

### 3.1. Comparison of the Performance of the ISS and NISS for the Prediction of the Outcomes in Patients with Thoracic Trauma

#### 3.1.1. Pneumonia

**Figure 2:**
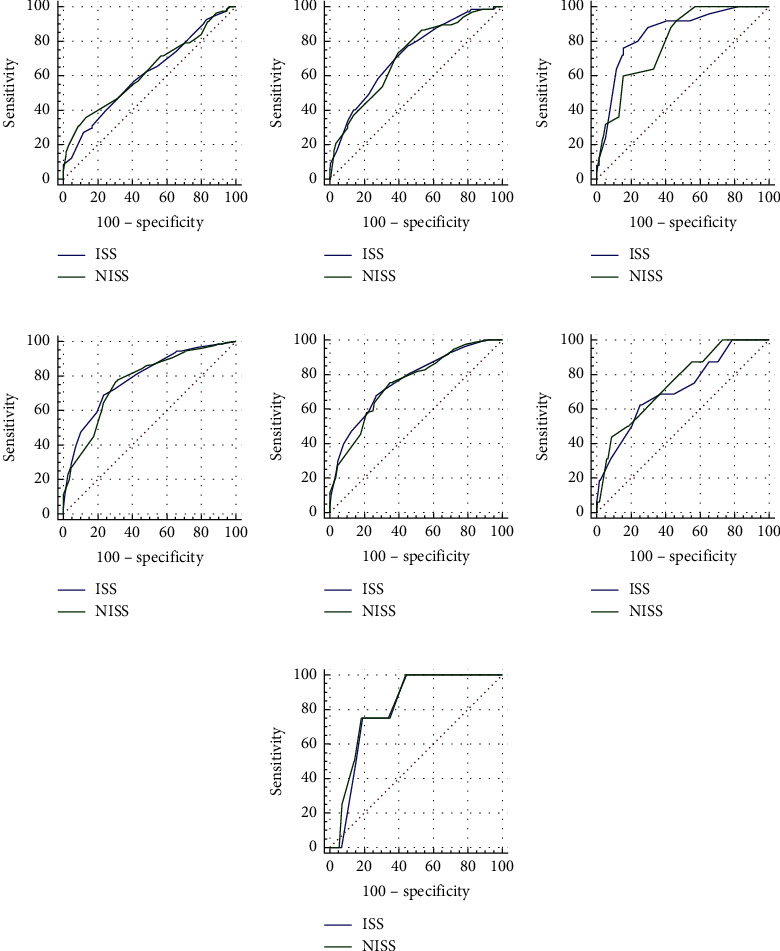
Comparison of the receiver operating characteristic (ROC) curves of the injury severity score (ISS) and new injury severity score (NISS) for the prediction of (a) pneumonia, (b) respiratory failure, (c) in-hospital tracheal intubation, (d) extended length of hospital stay, (e) intensive care unit (ICU) admission, (f) prolonged ICU stay, and (g) death.

We did not find a statistically significant difference in discrimination between the ISS and NISS for predicting pneumonia (AUC: 0.609 versus 0.628; *p*=0.5385). The calibration of the ISS was superior to that of the NISS (H-L: 2.411 versus 17.204). In addition, the calibration of the NISS was statistically insufficient (*p*=0.016) (Figure 2(a) and Table 4).

#### 3.1.2. Respiratory Failure

There was no statistically significant difference between the ISS and NISS in their discrimination for predicting respiratory failure (AUC: 0.721 versus 0.712; *p*=0.7907). The ISS showed a better calibration than the NISS (H-L: 1.982 versus 6.162) (Figure 2(b) and Table 4).

#### 3.1.3. In-Hospital Tracheal Intubation

There did not exist a statistically significant difference in discrimination between the ISS and NISS when predicting in-hospital tracheal intubation (AUC: 0.848 versus 0.795; *p*=0.1722). The calibration of the ISS was better than that of the NISS (H-L: 6.885 versus 11.294) (Figure 2(c) and Table 4).

#### 3.1.4. Extended Length of Hospital Stay

A statistically significant difference in discrimination between the ISS and NISS for predicting the extended length of hospital stay was not found (AUC: 0.784 versus 0.767; *p*=0.4563). The ISS was with a better calibration than the NISS (H-L: 1.699 versus 9.612) (Figure 2(d) and Table 4).

#### 3.1.5. ICU Admission

No statistically significant difference was observed in discrimination between the ISS and NISS for the prediction of ICU admission (AUC: 0.763 versus 0.750; *p*=0.5841). The calibration of the ISS was better than that of the NISS (H-L: 2.836 versus 10.781) (Figure 2(e) and Table 4).

#### 3.1.6. Prolonged ICU Stay

There was no statistically significant difference between the ISS and NISS in their discrimination to predict prolonged ICU stay (AUC: 0.716 versus 0.750; *p*=0.6089). The NISS outperformed the ISS in terms of calibration (H-L: 3.958 versus 8.067) (Figure 2(f) and Table 4).

#### 3.1.7. Death

The discrimination did not differ statistically significantly between the ISS and NISS in predicting death (AUC: 0.804 versus 0.818; *p*=0.9069). The calibration of the NISS was better than that of the ISS (H-L: 4.180 versus 7.919) (Figure 2(g) and Table 4).

## 4. Discussion

Thoracic trauma can cause respiratory complications and poor prognoses. Accurately evaluating trauma severity and predicting the occurrence of complications and poor prognoses are crucial for developing effective treatment plans for patients while also optimizing the allocation of medical resources. In the present study, we found that the ISS and NISS were both well suited for injury evaluation in patients with thoracic trauma. For predicting the outcomes including complications (pneumonia and respiratory failure) and poor prognoses (in-hospital tracheal intubation, extended length of hospital stay, ICU admission, prolonged ICU stay, and death) of thoracic trauma patients, the ISS and NISS were not statistically significantly different in discrimination. The calibration of the ISS and NISS in predicting different outcomes varied. The ISS demonstrated better calibration than the NISS for predicting pneumonia, respiratory failure, in-hospital tracheal intubation, extended length of hospital stay, and ICU admission, whereas the NISS showed better calibration for predicting prolonged ICU stay and death.

Falls are the second leading cause of death from unintentional injuries, and most fall-related injuries affect the head, spine, and chest [32]. Traumatic injuries, particularly to the chest, have increased steadily as a result of the increase in traffic accidents [3]. Assessment of the mechanisms of injury in the present study showed that the two most frequent mechanisms of injury were falls and traffic accidents, which was in accordance with the findings from another study including patients with multiple traumas [33].

The more severe the patient's injuries are, the greater the risks of complications and poor prognoses. In this study, the ISS and NISS were significantly greater in patients with complications and poor prognoses than in those without complications or poor prognoses. These findings indicated that the ISS and NISS could well reflect the severity of the trauma, so the two scoring systems were both suitable for injury evaluation in patients with thoracic trauma.

Although the ISS was developed to predict death, it has also been used to model many other outcomes [34]. Since the NISS was proposed, many studies have compared the ability of the ISS and NISS to predict the outcomes of trauma patients. However, the results have been controversial. Some studies have shown that the NISS is superior to the ISS, while others have suggested that the ISS and NISS have similar predictive abilities [17–19, 35–37]. To comprehensively explore the scope of optimal application of the ISS and NISS, it is important to conduct studies that compare the performance of the ISS with that of the NISS in accordance with certain parameters related to trauma outcomes, such as age, injury mechanism, and body region injured [34].

Several studies exploring the ability of the ISS and NISS to predict outcomes according to specific body regions have been performed. Lavoie et al. [18] reported that the NISS outperformed the ISS in terms of both discrimination and calibration for predicting ICU admission in patients with moderate to severe head injuries. Lavoie et al. [34] also found that the NISS showed advantages over the ISS both in discrimination and calibration for predicting in-hospital death in patients with head/neck injuries, facial injuries, thoracic injuries, and abdominal injuries. Jang et al. [38] reported that the AUCs of the NISS were larger than those of the ISS when predicting laparotomy and in-hospital mortality in patients with abdominal trauma, while the AUC of the ISS was larger than that of the NISS when predicting long hospital stay.

When predicting in-hospital mortality in patients with thoracic injuries, Lavoie et al. [34] reported the superiority of the NISS over the ISS in terms of both discrimination and calibration, but the NISS was not much better than the ISS in terms of discrimination (for the AUC, NISS: 0.809, ISS: 0.802, and *p*=0.04; for the H-L statistic, NISS: 20.9 and ISS: 50.1). Another study showed that the AUC of the NISS was larger than that of the ISS for predicting mortality in patients with thoracic trauma, but there was no statistical comparison of the discrimination between the two scoring systems (for the AUC, NISS: 0.876 and ISS: 0.867) [39]. These results were similar to our results, which showed that the calibration of the NISS was better than that of the ISS and that the NISS had a larger AUC without a statistical difference in discrimination compared with the ISS (for the AUC, NISS: 0.818, ISS: 0.804, and *p*=0.9069; for the H-L statistic, NISS: 4.180 and ISS: 7.919). In these three studies, both the ISS and NISS showed good capability according to the AUC (0.8 < AUC < 0.9) for the prediction of mortality.

Hu et al. [40] reported that the AUC of the NISS was significantly larger than that of the ISS for predicting respiratory failure in patients with chest trauma (for the AUC, NISS: 0.91, ISS: 0.84, and *p*=0.045), but the calibration analysis was not reported. In our study, the results did not support this finding, as the discrimination was not statistically significantly different between the ISS and NISS; moreover, the AUC of the ISS was larger than that of the NISS and the calibration of the ISS was better than that of the NISS (for the AUC, ISS: 0.721, NISS: 0.712, and *p*=0.7907; for the H-L statistic, ISS: 1.982 and NISS: 6.162).

In addition to death and respiratory failure, the other five outcomes, namely, pneumonia, in-hospital tracheal intubation, extended length of hospital stay, ICU admission, and prolonged ICU stay, are also common in patients with thoracic trauma. However, to date, few studies have been conducted to compare the performance of the ISS and NISS for predicting these five outcomes in patients with thoracic trauma. To close the gap in the literature, our study compared the performance of the ISS and NISS in predicting these outcomes. We found that for predicting these five outcomes in the present study, the ISS and NISS did not statistically significantly differ in terms of discrimination.

To our knowledge, this is the first study to compare the performance of the ISS and NISS for predicting pneumonia in trauma patients. However, for predicting pneumonia in patients with thoracic trauma, both the ISS and NISS had poor predictive ability (0.6 < AUC < 0.7) (for the AUC, ISS: 0.609 and NISS: 0.628). These findings indicate that when predicting pneumonia, in addition to the ISS and NISS, which reflect the anatomical injury severity, the importance of exploring the influence of other risk factors, such as age, sex, physiologic conditions, and comorbidities, should be considered.

For predicting in-hospital tracheal intubation in patients with thoracic trauma, this study showed that although the ISS and NISS were not statistically significantly different in terms of discrimination, the AUC of the ISS represented good predictive power, whereas the AUC of the NISS represented fair predictive power, and in addition, the calibration of the ISS was better than that of the NISS (for the AUC, ISS: 0.848, NISS: 0.795, and *p*=0.1722; for the H-L statistic, ISS: 6.885 and NISS: 11.294). Studies comparing the ability of these two scoring systems to predict in-hospital tracheal intubation in trauma patients are scarce. A study on trauma patients who were admitted to the ICU reported that the NISS outperformed the ISS for the prediction of the need for intubation (for the AUC, NISS: 0.863, ISS: 0.788, and *p* < 0.05; for the H-L statistic, NISS: 9.1 and ISS not reported) [41].

For predicting extended length of hospital stay in thoracic trauma patients, the results of the present study showed that the ISS was not statistically significantly different from the NISS in terms of discrimination but had a larger AUC than that of the NISS, and the ISS had a better calibration. These findings were similar to those reported by Tamim et al. [19]. They reported that the ISS was statistically better than the NISS in terms of discrimination and had a better calibration for predicting the length of hospital stay of ≥10 days in trauma patients (for the AUC, ISS: 0.72, NISS: 0.70, and *p*=0.03; for the H-L statistic, ISS: 11.0 and NISS: 15.78). In contrast, Balogh et al. [22] demonstrated that the NISS was a better predictor of the extended (≥10 days) length of hospital stay than the ISS in patients with multiple orthopaedic injuries.

In this study, although no statistically significant difference was found between the ISS and NISS in discrimination for the prediction of ICU admission in thoracic trauma patients, the ISS had a larger AUC and better calibration. A study conducted by Tamim et al. [19] also showed that the AUC of the ISS was larger than that of the NISS and that the ISS had a better calibration for predicting ICU admission in trauma patients (for the AUC, ISS: 0.81, NISS: 0.70, and *p*=0.0001; for the H-L statistic, ISS: 7.83 and NISS: 39.07). Lavoie et al. [18] reported that there was no statistically significant difference in discrimination between the ISS and NISS for predicting ICU admission in trauma patients; the AUC of the ISS was larger than that of the NISS, but the NISS showed an improved calibration compared to the ISS (for the AUC, ISS: 0.843, NISS: 0.839, and *p*=0.08; for the H-L statistic, ISS: 611 and NISS: 309). However, two other studies involving severe blunt trauma patients and patients with multiple orthopaedic injuries found that the NISS was superior to the ISS in predicting ICU admission [17, 22].

Our results indicated that the NISS was not statistically significantly different from the ISS in discrimination for predicting prolonged ICU stay among patients with thoracic trauma, but the NISS had a larger AUC and superior calibration (for the AUC, ISS: 0.716, NISS: 0.750, and *p*=0.6089; for the H-L statistic, ISS: 8.067 and NISS: 3.958). The findings of this study were similar to those of two other studies. Li et al. [17] reported that the NISS had a larger AUC and better calibration than the ISS for predicting prolonged ICU stay (>14 days) in severe blunt trauma patients (for the AUC, NISS: 0.772, ISS: 0.760, and *p*=0.0460; for the H-L statistic, NISS: 38.82 and ISS: 43.58). Harwood et al. [35] demonstrated that there was no statistically significant difference between the ISS and NISS in discrimination for predicting the length of ICU stay of >7 days in trauma patients, but the ISS had a larger AUC than the NISS (for the AUC, ISS: 0.763 and NISS: 0.762). Our study, along with the other two studies, demonstrated that both the ISS and NISS had fair predictive ability (0.7 < AUC < 0.8) for prolonged ICU stay, although one of the studies considered the length of ICU stay of >7 days as prolonged ICU stay.

At present, the ISS is considered an essential measurement in trauma evaluation globally and has long been employed for trauma research and benchmarking [17]. As a modification of the ISS, the NISS has two main advantages over the ISS: from a clinical perspective, the NISS is more logical than the ISS because it assigns the same importance to all injuries, even if they exist within the same body region; from a practical perspective, the NISS is easier to calculate and more effective compared to the ISS, as the NISS does not necessitate the division of AIS codes into body regions [18, 42]. Meanwhile, two scenarios may cause the overestimation of injury severity for NISS when a patient has two injuries in the same body region: adjacent injuries potentially due to the same impact and a secondary injury resulting from a primary injury. The advantages and disadvantages of the NISS may lead to uncertain results when comparing the performance of the ISS and NISS for predicting the outcomes of trauma patients in different subsets. The findings of our study and previous studies support this viewpoint, as the predictive performance of the ISS and NISS varies among different subgroups. Thus, it has recently been suggested that the ISS should not be replaced by the NISS [36]. Therefore, in clinical practice, the ISS and NISS should be selected based on their optimal scope of application. Further research is needed to explore the best application scope of ISS and NISS in more detail.

### 4.1. Strengths and Limitations

Compared to studies previously conducted to explore the predictive performance of the ISS and NISS not only in thoracic trauma patients but also in other subgroups or in the general trauma population, this study included a broad range of complications and prognostic indices. This is the first strength of this study. Second, as far as we know, this study is the first to elucidate and compare the predictive performance of the two scoring systems for pneumonia, which is one of the most common complications of thoracic trauma, thereby bridging the gap in the literature. Third, when assessing the predictive performance of the two scoring systems, calibration was calculated in addition to discrimination in this study, whereas many other studies only calculated discrimination. The results of this research provided a more thorough depiction of the predictive efficacy of the two scoring systems.

Our study has several limitations. First, this was a retrospective study, which meant that there were potential biases and limitations inherent to this type of study design. Second, the sample size included in the current study was not overly large, and the patients were from a single center, which may limit the generalizability of our findings.

## 5. Conclusion

This study revealed that the ISS and NISS of thoracic trauma patients with complications and poor prognoses were significantly greater than those of thoracic trauma patients without complications and poor prognoses. Therefore, both the ISS and NISS are suitable for injury evaluation in patients with thoracic trauma. In terms of predicting complications and poor prognoses in thoracic trauma patients, there was no statistically significant difference in discrimination between the ISS and NISS. Except for their ability to predict pneumonia, both scoring systems had a fair or good ability to predict other complications and poor prognoses. For predicting pneumonia, both the ISS and the NISS showed poor predictive ability, and the ISS had a smaller AUC than the NISS but better calibration. The ISS had a larger AUC and better calibration than the NISS in predicting respiratory failure, in-hospital tracheal intubation, extended length of hospital stay, and ICU admission, respectively. The NISS had a larger AUC and better calibration than the ISS in predicting prolonged ICU stay and death, respectively. These results can provide a reference for the clinical application of these two scoring systems for evaluating injury and predicting complications and poor prognoses in patients with thoracic trauma.

## Figures and Tables

**Table 1:** Description of the patients.

Characteristic/outcome	Number (%)/mean (SD)/median (IQR)
All patients	310
Sex, *n* (%)	
Male	231 (74.5%)
Female	79 (25.5%)
Age (years), mean (SD)	49.47 (12.15)
Score, median (IQR)	
ISS	13 (9–17)
NISS	17.5 (11–22)
Mechanism, *n* (%)	
Fall	174 (56.1%)
Traffic accident	84 (27.1%)
Others	52 (16.8%)
Pneumonia, *n* (%)	
Yes	81 (26.1%)
No	229 (73.9%)
Respiratory failure, *n* (%)	
Yes	67 (21.6%)
No	243 (78.4%)
In-hospital tracheal intubation, *n* (%)	
Yes	25 (8.1%)
No	285 (91.9%)
Length of hospital stay (day), median (IQR)	13 (8, 19)
Extended length of hospital stay, *n* (%)	
Yes	202 (65.2%)
No	108 (34.8%)
Admission to the ICU, *n* (%)	
Yes	198 (63.9%)
No	112 (36.1%)
Length of ICU stay (day), median (IQR)	4 (2, 7)
Prolonged ICU stay, *n* (%)	
Yes	16 (8.1% of the 198 patients admitted to the ICU)
No	182 (91.9% of the 198 patients admitted to the ICU)
Death, *n* (%)	
Yes	4 (1.3%)
No	306 (98.7%)

SD, standard deviation; IQR, interquartile range; ISS, injury severity score; NISS, new injury severity score; ICU, intensive care unit.

**Table 2:** Comparison of the ISS and NISS between different subgroups.

Outcome	Patients (n)	ISS, median (IQR)	*p* value	NISS, median (IQR)	*p* value
Pneumonia			0.003		0.001
Yes	81	14 (9, 21)		21 (14, 27)	
No	229	11 (9, 16)		17 (11, 22)	
Respiratory failure			<0.001		<0.001
Yes	67	16 (13, 21)		22 (18, 27)	
No	243	11 (9, 16)		17 (10, 22)	
In-hospital tracheal intubation			<0.001		<0.001
Yes	25	21 (17.5, 22.5)		24 (21, 31.5)	
No	285	11 (9, 16)		17 (11, 22)	
Extended length of hospital stay			<0.001		<0.001
Yes	202	14 (10, 20)		21 (17, 24)	
No	108	9 (5, 11)		12 (6, 17)	
Admission to the ICU			<0.001		<0.001
Yes	198	14 (10, 20)		21 (16.5, 24)	
No	112	9 (5, 13)		14 (6, 18.75)	
Prolonged ICU stay			0.004		0.001
Yes	16	20.5 (13.25, 24)		25.5 (21.25, 34)	
No	182	14 (10, 20)		21 (14, 22.5)	
Death			0.036		0.028
Yes	4	20.5 (15.5, 21)		25.5 (21.75, 28.5)	
No	306	13 (9, 17)		17 (11, 22)	

ISS, injury severity score; NISS, new injury severity score; IQR, interquartile range; ICU, intensive care unit.

**Table 3:** ROC analysis of the ISS and NISS for predicting the outcomes.

	AUC (95% CI)	Cutoff (95% CI)	Sensitivity (95% CI)	Specificity (95% CI)	PPV (95% CI)	NPV (95% CI)	PLR (95% CI)	NLR (95% CI)	Youden index (95% CI)
Pneumonia									
ISS	0.609 (0.552–0.664)	>13 (>4–>20)	0.568 (0.453–0.678)	0.590 (0.523–0.654)	0.329 (0.277–0.385)	0.794 (0.746–0.835)	1.384 (1.083–1.768)	0.733 (0.558–0.962)	0.157 (0.064–0.230)
NISS	0.628 (0.571–0.681)	>22 (>7.4–>25)	0.358 (0.254–0.472)	0.869 (0.818–0.910)	0.492 (0.383–0.601)	0.793 (0.763–0.819)	2.733 (1.755–4.257)	0.739 (0.623–0.876)	0.227 (0.123–0.318)
Respiratory failure									
ISS	0.721 (0.667–0.770)	>12 (>8–>14)	0.776 (0.658–0.869)	0.543 (0.478–0.607)	0.319 (0.280–0.361)	0.898 (0.847–0.933)	1.699 (1.408–2.050)	0.412 (0.260–0.653)	0.319 (0.199–0.408)
NISS	0.712 (0.658–0.762)	>18 (>16–>27)	0.731 (0.609–0.832)	0.605 (0.540–0.667)	0.338 (0.292–0.387)	0.891 (0.845–0.925)	1.851 (1.496–2.290)	0.444 (0.295–0.668)	0.336 (0.196–0.423)
In-hospital tracheal intubation									
ISS	0.848 (0.803–0.886)	>17 (>13–>20)	0.760 (0.549–0.906)	0.846 (0.798–0.886)	0.302 (0.233–0.380)	0.976 (0.952–0.988)	4.923 (3.470–6.984)	0.284 (0.141–0.571)	0.606 (0.402–0.717)
NISS	0.795 (0.746–0.839)	>17 (>14–>22)	0.920 (0.740–0.990)	0.537 (0.477–0.596)	0.148 (0.128–0.171)	0.987 (0.953–0.997)	1.986 (1.675–2.355)	0.149 (0.039–0.566)	0.457 (0.375–0.526)
Extended length of hospital stay									
ISS	0.784 (0.734–0.829)	>11 (>10–>14)	0.688 (0.619–0.751)	0.769 (0.678–0.844)	0.848 (0.796–0.888)	0.568 (0.512–0.624)	2.973 (2.082–4.244)	0.406 (0.323–0.511)	0.457 (0.349–0.545)
NISS	0.767 (0.716–0.813)	>14 (>11–>16)	0.777 (0.714–0.833)	0.685 (0.589–0.771)	0.822 (0.776–0.860)	0.622 (0.552–0.687)	2.469 (1.851–3.292)	0.325 (0.244–0.433)	0.462 (0.345–0.547)
ICU admission									
ISS	0.763 (0.711–0.809)	>11 (>10–>16)	0.677 (0.607–0.741)	0.732 (0.640–0.811)	0.817 (0.764–0.860)	0.562 (0.504–0.617)	2.527 (1.833–3.483)	0.441 (0.351–0.556)	0.409 (0.303–0.506)
NISS	0.750 (0.698–0.797)	>16 (>14–>19)	0.753 (0.686–0.811)	0.652 (0.556–0.739)	0.793 (0.745–0.833)	0.598 (0.530–0.663)	2.161 (1.657–2.819)	0.380 (0.288–0.501)	0.404 (0.291–0.504)
Prolonged ICU stay									
ISS	0.716 (0.648–0.778)	>19 (>16–>26)	0.625 (0.354–0.848)	0.747 (0.678–0.809)	0.179 (0.121–0.255)	0.958 (0.923–0.977)	2.473 (1.570–3.895)	0.502 (0.265–0.950)	0.372 (0.174–0.565)
NISS	0.750 (0.683–0.808)	>27 (>21–>33)	0.438 (0.198–0.701)	0.912 (0.861–0.949)	0.304 (0.175–0.475)	0.949 (0.923–0.966)	4.977 (2.407–10.290)	0.617 (0.399–0.952)	0.350 (0.194–0.485)
Death									
ISS	0.804 (0.755–0.846)	>19 (>13–>20)	0.750 (0.194–0.994)	0.810 (0.762–0.853)	0.049 (0.027–0.087)	0.996 (0.978–0.999)	3.957 (2.147–7.293)	0.308 (0.056–1.686)	0.560 (0.477–0.778)
NISS	0.818 (0.770–0.859)	>22 (>19–>27)	0.750 (0.194–0.994)	0.817 (0.769–0.859)	0.051 (0.028–0.090)	0.996 (0.979–0.999)	4.098 (2.219–7.568)	0.306 (0.056–1.672)	0.567 (0.480–0.794)

ROC, receiver operating characteristic; ISS, injury severity score; NISS, new injury severity score; AUC, area under the ROC curve; PPV, positive predictive value; NPV, negative predictive value; PLR, positive likelihood ratio; NLR, negative likelihood ratio; CI, confidence interval; ICU, intensive care unit.

**Table 4:** Comparison of the ISS and NISS for predicting the outcomes.

Outcome	ISS		NISS		*p* value^∗^
	AUC	H-L (*p* value)	AUC	H-L (*p* value)	
Pneumonia	0.609	2.411 (0.966)	0.628	17.204 (0.016)	0.5385
Respiratory failure	0.721	1.982 (0.982)	0.712	6.162 (0.521)	0.7907
In-hospital tracheal intubation	0.848	6.885 (0.549)	0.795	11.294 (0.126)	0.1722
Extended length of hospital stay	0.784	1.699 (0.989)	0.767	9.612 (0.212)	0.4563
ICU admission	0.763	2.836 (0.944)	0.750	10.781 (0.148)	0.5841
Prolonged ICU stay	0.716	8.067 (0.427)	0.750	3.958 (0.861)	0.6089
Death	0.804	7.919 (0.441)	0.818	4.180 (0.759)	0.9069

ISS, injury severity score; NISS, new injury severity score; AUC, area under the ROC curve; H-L, Hosmer‒Lemeshow statistic; ICU, intensive care unit; ROC, receiver operating characteristic. ^∗^Comparison of AUC between the ISS and NISS.

## Data Availability

The data used to support the findings of the study are available from the corresponding author upon reasonable request.
